# Disturbed homocysteine metabolism is associated with cancer

**DOI:** 10.1038/s12276-019-0216-4

**Published:** 2019-02-21

**Authors:** Tauheed Hasan, Reetika Arora, Aniket Kumar Bansal, Reshmee Bhattacharya, Gurumayum Suraj Sharma, Laishram Rajendrakumar Singh

**Affiliations:** 0000 0001 2109 4999 grid.8195.5 Dr. B. R. Ambedkar Center for Biomedical Research, University of Delhi, Delhi, 110 007 India

**Keywords:** Cancer metabolism, Cancer prevention

## Abstract

Hyperhomocysteinemia/Homocysteinuria is characterized by an increased level of toxic homocysteine in the plasma. The plasma concentration of homocysteine is 5–15 μmol/L in healthy individuals, while in hyperhomocysteinemic patients, it can be as high as 500 μmol/L. While increased homocysteine levels can cause symptoms such as osteoporosis and eye lens dislocation, high homocysteine levels are most closely associated with cardiovascular complications. Recent advances have shown that increased plasma Hcy is also a fundamental cause of neurodegenerative diseases (including Alzheimer’s disease, Parkinson’s disease, and dementia), diabetes, Down syndrome, and megaloblastic anemia, among others. In recent years, increased plasma homocysteine has also been shown to be closely related to cancer. In this review, we discuss the relation between elevated plasma Hcy levels and cancer, and we conclude that disturbed homocysteine metabolism is associated with cancer. Future clinical perspectives are also discussed.

## Cancer: Links with homocysteine

Cancer can be added to the wide range of diseases known to be associated with elevated blood levels of the small amino acid homocysteine. Abnormally high levels of this compound are already known to contribute to conditions including cardiovascular problems, neurodegenerative diseases, neural tube defects, Down’s syndrome, diabetes and megaloblastic anemia. This review, by Laishram R. Singh and colleagues at the University of Delhi, India, concludes that disturbed homocysteine metabolism is associated with many forms of human cancer. The authors discuss a range of genetic, epigenetic and environmental factors that may be involved in the cause and effect relationships between homocysteine metabolism and cancer. It is particularly interesting that low folate (vitamin B9) levels result in high homocysteine levels, and vice versa. Further research may yield insights leading to new forms of cancer treatment and diagnosis.

## Introduction

**Fig. 1:**
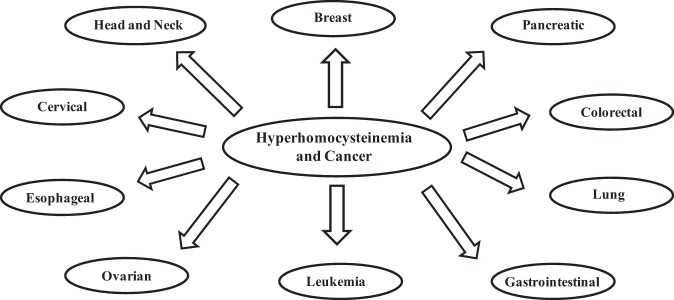
Association between hyperhomocysteinemia and various cancer types

Homocystinuria is an inborn error in the metabolic pathways of sulfur-containing amino acids and is characterized by an increase in the level of toxic homocysteine (Hcy) in the serum^1^. Mutations in cystathionine beta synthase (CBS), an enzyme present at the branch point between the trans-sulfuration and remethylation pathways, are the basic cause of homocysteinemia. The term “hyperhomocysteinemia” is also used to describe the elevated Hcy serum level due to other genetic (CBS-independent) and environmental factors^2^. In a normal, healthy individual, the serum Hcy level is between 5–15 μM, but it can increase to 50 μM in mild cases and to 500 μM in severe cases of homocysteinemia (de Koning, Werstuck et al. 2003). This hyperhomocysteinemic condition is closely related to many disease conditions (Table 1). It is believed that increased homocysteine levels lead to various cardiovascular complications (Table 1)^3,4^ If the Hcy level is left uncontrolled, patients ultimately die of stroke^5^. Further studies have also revealed that elevated plasma Hcy level is one of the key factors associated with neurodegeneration, diabetes, Down syndrome, neural tube defects, and megaloblastic anemia (see Table 1)^2,4,6–9^. Hyperhomocysteinemia has also been connected to various other clinical complications, including ectopic lentis, scoliosis, megaloblastic anemia, knocked knees, long limbs, and arachnodactyly, among others (Table 1)^4,10–12^. Recent advances have proven that there is a close link between hyperhomocystinuria and cancer (see Fig. 1). First, higher levels of plasma homocysteine have been observed cancer patients, and venous thromboembolism (VTE) is the second most common cause of death in cancer patients. Second, several polymorphisms in the enzymes involved in the Hcy detoxification pathways (the trans-sulfuration and remethylation) have close clinical ties to several cancer types^13–23^. Third, folate, which is pivotal for cell proliferation, has an inverse relation with Hcy. Fourth, Hcy has also been proposed as a potential tumor biomarker for a variety of cancers^24^. In this review, we have systematically discussed these important key events in detail and revealed that defects in Hcy metabolism may lead to cancer. Future clinical perspectives have also been described.

**Table 1:** Homocysteinemia and its associated disorders

Complication	Associated diseases	References
Cardiovascular diseases	Thromboembolism	^130, 131^
	Coronary artery	^132^
	Atherosclerosis	^133, 134^
	Vascular dementia	^135, 136^
	Congenital heart defects	^137, 138^
	Stroke	^139, 140^
Neurodegeneration	Alzheimer’s	^6^
	Parkinson	^141, 142^
	Schizophrenia	^143, 144^
	Dementia	^145, 146^
	Depression	^147, 148^
Diabetes	—	^149, 150^
Down’s syndrome	—	^151^
Megaloblastic anemia	—	^152^
Other diseases	Neural tube defects	^55^
	Nonsyndromic oral cleft	^153^
	Ectopic lentis	^6, 139, 140^
	Scoliosis	^154^
	Knocked knees	^154^
	Long limbs	^154^
	Arachnodactyly	^154^
Cancer	Refer to Fig. 3

## I (a) Low folate levels help build plasma Hcy

**Fig. 2:**
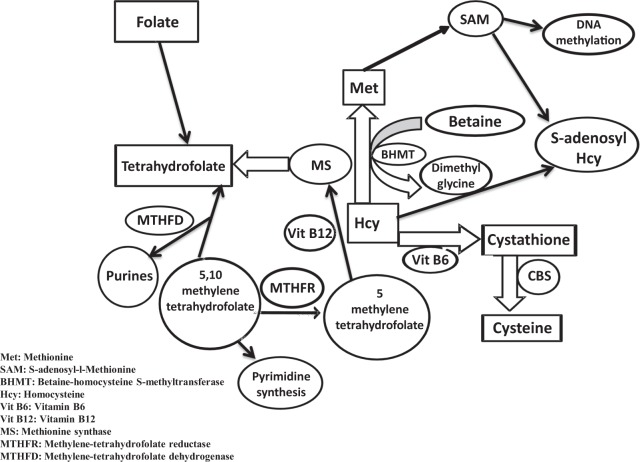
Folate metabolism is linked to the homocysteine metabolic pathway

Homocysteine is a sulfur-containing, nonprotein, toxic amino acid found in the pathway for the interconversion of two amino acids: methionine and cysteine. Homocysteine is metabolized via two different pathways: remethylation and trans-sulfuration^25,26^. When there is an excess of cellular methionine, the trans-sulfuration pathway plays a crucial role in Hcy metabolism, converting Hcy to cystathionine via CBS, which requires pyridoxal 5′-phosphate as a co-factor^25,27^. When the cellular methionine level is low, Hcy is remethylated back to methionine in a betaine- or folate-dependent reaction. In the betaine-dependent pathway, the enzyme betaine-homocysteine S-methyltransferase (BHMT)^28,29^ catalyzes the incorporation of a methyl group from betaine into homocysteine to form methionine. In the folate-dependent pathway, Hcy acquires a methyl group from N-5-methyltetrahydrofolate with the help of 5-methyltetrahydrofolate-homocysteine methyltransferase (MTR) (also known as methionine synthase). Methionine synthase requires vitamin B12 for its functionality, and the reaction also involves recycling of tetrahydrofolate (from N-5-methyltetrahydrofolate), which may eventually be used for nucleotide biosynthesis^30^. Methionine synthase, therefore, couples the folate and Hcy metabolism pathways (Fig. 2). Since the generation of tetrahydrofolate depends on the input of exogenous folate for folate metabolism (as outlined in Fig. 2), low folate levels ultimately result in substrate limitation for methionine synthase, thereby affecting the remethylation pathway. Thus, low folate levels result in a high plasma Hcy concentration and vice versa.

Many factors that affect the folate level have also been found to disturb the Hcy level. For instance, diets deficient in folate, cobalamin, and vitamin B6^31^ and use of anti-folate drugs (including anticonvulsants and other neurological drugs^32^) directly increase the plasma Hcy level. Drugs that elevate the Hcy level (e.g., laxatives, diuretics, birth control pills, anti-inflammatory drugs, immune suppressants) also reduce the folic acid levels^33–36^. Other conditions, including alcohol consumption^13,18^, smoking, diabetes, and psoriasis^37^, among others, are responsible for reducing the plasma folate level by affecting the folate level. Therefore, it is important to take folate supplements to restore the depleted Hcy pool.

## I (b) Low plasma folate levels lead to cancer predisposition

Folate is not only involved in nucleotide biosynthesis but also required for the conversion of deoxyuridine monophosphate (dUMP) into thymidine monophosphate^38^. Under normal conditions, thymidylate synthetase (TYMS) converts dUMP into thymidine monophosphate using 5,10-methylenetetrahydrofolate (derived from folate) as a methyl group donor. If folate is limiting, dUMP accumulates because its key methyl donor, 5,10-methylenetetrahydrofolate, is absent. These conditions lead to an imbalance in the deoxyribonucleotide pool, and, consequently, there is excessive incorporation of uracil into DNA instead of thymine; this defect is normally repaired by the enzyme uracil DNA glycosylase, which removes the misincorporated uracil from the DNA strand^39^. When the folate concentration is disturbed (due to increased Hcy levels) the DNA glycosylase fails to cope with the DNA repair burden. This situation leads to chromosomal damage, which may then lead malignant transformation in cells. Furthermore, excision repair of uracil residues 12 base pairs apart can lead to double strand breaks, which may increase DNA instability due to relaxed DNA supercoiling and chromosomal remodeling, both of which can cause an increase in malignant transformation. Chromosomal aberrations are also associated with inappropriate differentiation and morphology of lineage-specific cells, features often associated with tumors^40^.

**Fig. 3:**
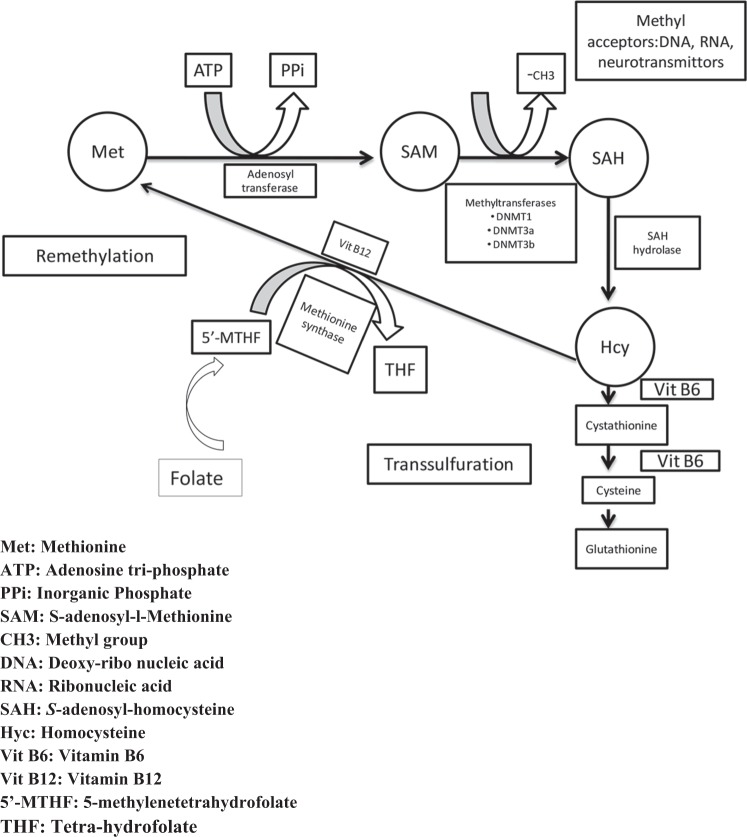
Low folate levels result in Hypomethylation

Low plasma folate levels are also linked to cancer is via DNA methylation. DNA methylation is an epigenetic modification that is critical for normal genome regulation and development. Indeed, it is Hcy that is recycled to methionine with the help of methionine synthase. DNA methylation is carried out with the help of a methyl donor, S-adenosyl-l-methionine (SAM), which is obtained from methionine via an ATP-dependent reaction catalyzed by S-adenomethyl synthetase^41^. DNA methylation is jointly carried out by three types of DNA methyltransferases (DNMTs)—DNMT1, DNMT3a, and DNMT3b on SAM (Fig. 3). Since SAM is generated from 5-methyltetrahydrofolate (5′-MTHF) as shown in Fig. 3, low folate levels limit the substrate availability for methionine synthase, thereby resulting in DNA hypomethylation. DNA hypomethylation leads to decondensation of pericentromeric heterochromatin and the activation of retrotransposon elements^42^. Global genomic hypomethylation has been found in many types of cancer, including prostate metastatic tumors, chronic lymphocytic tumors, and hepatocellular carcinoma. Regional hypomethylation of DNA sequences is also often observed during the early stages of tumorigenesis and in abnormal nonneoplastic tissue, such as hyperplasia^43^.

## I (c) Cancer patients have high plasma Hcy levels

As mentioned in the earlier sections, there is an inverse relation between plasma Hcy and folate. In cancer patients, the plasma folate level is expected to be low because tumor cells must draw folate from the blood for de novo purine synthesis^44,45^. Interestingly, as shown in Fig. 1, hyperhomocystinuria is associated with several types of cancer. It is also clear from the information in this figure that the causative relationship between homocysteine toxicity and cancer is independent of the organ/tissue and the type of cancer. Table 2 shows that all cancer types in the advanced stage exhibit high plasma Hcy levels, while there was no significant change in plasma Hcy levels in early stage cancer. Furthermore, once patients are subjected to surgery or chemotherapy, there is also a sharp increase in the plasma Hcy level, leading to a higher frequency of thromboembolic events. Because most commonly used clinical chemotherapeutic agents (such as alkylating agents, antimetabolites, methotrexate, hormones, and antagonists) are anti-folate drugs^46^, their use causes a decrease in the plasma folic acid concentration. In another development, it has also been shown that older cancer patients are at a higher risk of developing hyperhomocysteinemia than are younger patients^47^.

**Table 2:** Polymorphisms detected in genes involved in homocysteine metabolism

Gene	Polymorphisms	Amino acid change	Cancer type	OR values	References
Methylene-tetrahydrofolate reductase (MTHFR)	677C- > T	A226V	Endometrial carcinoma	1.10	^155^
			Esophageal squamous cell carcinoma (SCC)	1.47	^156^
			Breast cancer	1.00^a^/1.12/1.00^a^	^15, 99, 157^
			Acute lymphocytic leukemia (ALL)	0.99/0.23	^87^
			Prostate cancer	0.78	^158^
			Colorectal cancer	1.78/1.00^a^/0.76	^85, 159– 161^
	1298A- > C	E443A	Prostate cancer	0.58	^79^
			Acute myeloid leukemia	0.33/1.00	^87, 162^
			Endometrial cancer	0.88	^155^
	1793G- > A	R1793E	Colorectal cancer	0.17	^163^
			Acute myeloid leukemia	1.00	^162^
Methionine synthase reductase (MTRR)	66A- > G	I22M	Leukemia	1.00^a^	^164, 165^
			Colorectal cancer	2.77/1.07	^18, 165, 166^
			Gastric cancer	0.74/1.39	^167, 168^
			Breast cancer	4.45	^169^
Methionine synthase (MTR)	2756A- > G	D919G	Head and neck carcinoma	1.10	^170^
			Colorectal cancer	1.03/0.65/2.04	^161, 170, 171^
			Lung cancer	1.34	^172^
			Hepatocellular carcinoma	1.01	^170, 173^
			Cervical cancer	0.27	^14^
			Glioblastoma multiforme	1.00^a^	^174^
			Breast cancer	1.00^a^	^99^
			Squamous cell carcinoma	1.00^a^	^98^
			Gastric cancer	1.06/1.35	^168, 170, 175^
			Pancreatic cancer	1.08/3.35	^170, 176^
Methylene-tetrahydrofolate dehydrogenase (MTHFD1)	1958G- > A	A653G	Gastric cancer	2.05	^102^
			Leukemia	0.80	^177^
	401G- > A	R134K	Gastric cancer	1.43	^102^
			Leukemia	0.89	^177^
			Ovarian cancer	0.97	^178^
Betaine-homocysteine methyltransferase (BHMT)	742G- > A	R239Q	Squamous cell carcinoma	1.07	^179^
			Breast cancer	0.98/0.12	^111, 180^
			Uterine carcinoma	0.64	^181^
			Ovarian cancer	1.01	^182^
			Colorectal adenoma	1.09	^183^
			Liver cancer	0.98	^184^
	595G- > A	G199S	—		
	716G- > A	Q239R	—		
	1218G- > T	Q406H	—		
Cystathionine β-synthase (CBS)	833T- > C	I278T	—		
	699C- > T	Y233Y	—		
	1080C- > T	A360A	—		
	572C- > T	T191M	—		
	139C- > T	S466L	—		
	502G- > A	V168M	—		
	797G- > A	R266K	—		
	1150A- > G	K384E	—		
	341C- > T	A114V	—		
	919G- > A	G307S	—		
TCN 2	776 G > C	R259P	Colorectal adenoma	0.753	^183^
			Colorectal cancer	1.137	^185^
			Glioblastoma	1.028	^174^
			Primary central nervous system lymphoma	1.338	^186^
			Ovarian cancer	1.389	^182^
TYMS	TS 3́-UTR	—	Esophageal cancer	0.73	^187^
		—	Stomach cancer	1.12	^187^
	TSER	—	Breast cancer	1.09	^187^

^a^Papers that reported no association have been given the value of 1.00

There is no clear explanation for why the Hcy levels vary between early and late stage cancer. However, we speculate that cells in the early stage might not secrete Hcy, as it facilitates the proliferation process of cancer cells^48^. Studies have shown that increased homocysteine levels lead to increased cellular proliferation in Caco-2 cell lines. This enhanced proliferation can be reversed by folate supplementation in the culture medium or by supplementation with its downstream metabolites, such as 5-MTHF^49^. However, advanced-stage cancer cells might secrete Hcy because a very high Hcy concentration might also be cytotoxic to the cancer cells. Therefore, it may be important for proliferating cells to maintain an optimum Hcy concentration. This speculation, however, requires further experimental validation.

## I (d) Cancer patients develop thromboembolisms due to Hcy toxicity

One major symptom of hyperhomocysteinemia is the formation of venous thromboembolism (VTE). VTE is the most frequent complication and second most common cause of death among cancer patients^50^. Advanced-stage cancer patients develop both hyperhomocysteinemia and VTE. Alternatively, in early cancer patients (without homocysteinuria), VTE is absent^51^. Indeed, the advanced-stage cancer patients have a greater risk for developing VTE, with a frequency of 5–15%^51,52^ (in comparison, the risk for the normal population is 0.1%). Postchemotherapy cancer patients (who are known to be at risk for homocystinuria) account for 13% of the total pool of VTE patients^53^. In postsurgery patients, their susceptibilities to embolism and thrombosis are increased three-fold and two-fold, respectively^54^. Use of central venous catheters and hormonal adjuvant therapy (e.g., Tamoxifen) also predisposes patients to VTE^51^ due to increased plasma Hcy levels. Thus, there is a close link between cancer and Hcy-induced development of VTE.

The mechanism underlying the cancer-related thrombosis induced by elevated Hcy^55^ is complex and not well understood. However, it has been thought to result from endothelial disturbances caused by the formation of Hcy-mediated free-radicals^56^. Hcy is a pro-oxidant, and the formation of Hcy-Hcy dimers and Hcy-protein adducts that help to generate free radicals are well established. Hcy can also form a more highly reactive compound called homocysteine thiolactone. Homocysteine thiolactone has been known to form covalent adducts with lysine or arginine residues in proteins, resulting in the formation of insoluble toxic protein aggregates or amyloids^2,57,58^. The deposition of such aggregates in the blood or heart may, therefore, impede normal heart function and physiology. Furthermore, modification of hemostatic proteins (via N-homocysteinylation or S-homocysteinylation) has also been reported to impede NO metabolism, which may cause biotoxicity in endothelial cells^59^. Hcy also inhibits thrombomodulin and Protein C-dependent inactivation of Factor V_a_;^60^ therefore, blood coagulation is enhanced in the presence of Hcy. Furthermore, Hcy limits the secretion of nitric oxide (NO), leading to increased platelet aggregation and decreased antithrombic activities in the endothelial cells^61,62^.

## I (e) Allelic polymorphisms in sulfur metabolism genes and associated risk of cancer

Various case control and cohort studies^63–70^ have shown that mutations and polymorphisms exist in genes involved in homocysteine metabolism (*MTHFR, CBS, MTRR, MTR, MTHFD, BHMT, TYMS, TCN 2*). Polymorphic alleles of these genes were found to be linked with neural tube defects^71^ and/or vascular thromboembolism^72,73^, which are symptoms of hyperhomocysteinemia. Recent studies have shown that these polymorphisms are also closely associated with different cancer types (Table 2). For instance, *MTHFR* has ~6375 polymorphisms, consisting of 650 deletions, 05 multiple base substitutions, 140 repeat variations, and 5580 SNPs^74^. Two common polymorphisms, a 677C- > T transition at codon 222 (Ala222Val)^19,27,63,73,75^ and a 1298A- > C transversion at codon 429 (Glu429Ala)^75,76^, have been reported to be associated with various cancer types, including endometrial carcinoma, esophageal squamous cell carcinoma (SCC), colon cancer, acute lymphocytic leukemia (ALL), and prostate cancer. In addition to 677C- > T and 1298A- > C, there is a third polymorphism, 1793G- > A, whose frequency is very low (~ 4.6% or less) and which is confined to colorectal cancer^77^. The 677C- > T polymorphism affects the protein’s catalytic activity and the 1298A- > C polymorphism affects its regulatory function^20,78^. Homozygotes (677 CC, ~ 60%) are more frequent than heterozygotes (677 CT, ~ 31%), but this pattern is reversed in the case of 1298 (1298AC, ~ 53% and 1298AA, ~ 31%). The 677TT and 1298CC homozygotes were found to have reduced prostate cancer risk, as the frequencies are very low (9 and 11%, respectively)^21,79^. The risk factor associated with the 677C- > T polymorphism has been found to depend on the type of cancer, as it confers a higher risk for endometrial carcinoma^80^(7), esophageal SCC^81^, and prostate cancer^82–84^, while it has little or no effect on the risk for colon cancer^14,85,86^ and acute lymphoid leukemia^87^. The variable behavior of 677C- > T in different cancer types indicates that the environment or genetic background might help to dictate the activity of the polymorphism. In this context^88^, a number of factors have been proposed, including folate status, methionine, and the effects of alcohol consumption, to be the risk factor connecting the 677C- > T polymorphism to colorectal cancer. Another possibility is that the 677C- > T polymorphism is dominant negative in some cancer types but not in others based on the functional effect of the polymorphism.

*MTRR* has ~9461 polymorphisms, out of which 1051 are deletions, 01 are multiple base substitutions, 315 are repeat variations, and 8094 are SNPs^89^. Out of this pool, only one polymorphism (*MTRR* A66G, Ile22Met)^14,18,90,91^ has been studied and found to be associated with leukemia and colorectal cancer. This leukemic polymorphism has an allelic frequency of 51% in white populations^87,90^ Comparison of the relationships between homozygosity and heterozygosity and colorectal cancer revealed that homozygotes (GG) have a three-fold higher risk compared with that of heterozygotes (AG)^14,92^. Since the leukemic allelic frequency is very high in the white population, it is important to investigate other populations to determine if genetic background affects the frequencies or functions of polymorphic *MTRR*.

Similarly, *MTR* has ~26150 polymorphisms, out of which 2643 are deletions, 06 are multiple base substitutions, 221 are repeat variations, and 23245 are SNPs^105^. One significant polymorphism (*MTR* A2756G; Asp919Gly) has been documented in *MTR*. This 2756A- > G variant is associated with head, esophageal and neck squamous cell carcinoma, colorectal adenoma, colorectal carcinoma, lung cancer, multiple myeloma, cervical cancer, uterine cancer, and glioblastoma multiforme^18,24,37,93–97^. This polymorphism is associated with a decreased risk for colorectal cancer^14,90^, and no correlation could be established between this *MTR* variant and the incidence of breast and upper gastrointestinal tract carcinomas^98,99^, suggesting that the severity of the effect of the polymorphism might depend on the type of cancer.

*MTHFD-*1 has ~16991 polymorphisms, out of which 1913 are deletions, 07 are multiple base substitutions, 215 are repeat variations, and 14856 are SNPs^100^. One polymorphism (1958G- > A; Ala653Gly)^97,101,102^ associated with acute lymphoid leukemia, which is located within the 10-formyl THF synthetase domain, has been reported^103^. No links between *MTHFD* variants and lung cancer risk could be established (Liu, Jin et al. 2008). Another polymorphism, (401G- > A; Arg134Lys), which is in the cyclohydrolase/dehydrogenase domain, was also reported by^102^, but it is associated with a low colon cancer risk.

*TYMS* expression is a highly regulated process that is modulated by unique tandem repeat sequences and significant polymorphisms in the 5́-UTR of the thymidylate synthase enhancer region (TSER) and in the 3́-UTR (TS1494del6b) of the gene^104^. *TYMS* has ~5892 polymorphisms, out of which 1078 are deletions, 05 are multiple base substitutions, 398 are repeat variations, and 4411 are SNPs^105^. The TSER variant is most commonly present as double- (2R) and triple- (3R) repeat sequences, although 4R, 5R, and 9R repeats also exist. 3R sequences show higher translational efficiency than 2R sequences. Homozygous 3R/3R subjects show higher TYMS protein expression and higher enzyme activity^106^. The second *TYMS* polymorphism identified is a 6 bp deletion/insertion at bp 1494 in the 3́-UTR of the *TYMS* gene. Several lines of evidence suggest that variation in the *TYMS* gene is associated with cancer risk. However, the risk factor associated with these polymorphisms depends on the type of cancer. In the cases of stomach, colorectal, and lung cancer, the TYMS enzyme activity and mRNA expression were found to be increased.

Transcobalamin II (TCN II) is a serum protein that transports vitamin B12 (cobalamin) from the ileum to other tissues. Vitamin B12 serves as an important molecule in the remethylation of methionine from Hcy and is important for the transformation of MTHF to THF. *TCN-2* has ~8291 polymorphisms, out of which 1183 are deletions, 02 are multiple base substitutions, 146 are repeat variations, and 6960 are SNPs^107^. One common polymorphism in the *TCN 2* gene is a G to C substitution (776 G > C, rs1801198) that results in replacement of a proline with an arginine^108^ In a recent meta-analysis, it was shown that subjects with the rs1801198 GG genotype had significantly lower concentrations of holotranscobalamin and higher Hcy levels compared to subjects with the rs1801198 CC genotype. This polymorphism has also been shown to be associated with different cancers, including colorectal cancer, ovarian cancer, glioblastoma, among others. However, most of the cancer types did not have close association, as indicated by OR values near or equal to 1. Colorectal cancer, ovarian cancer, and central nervous system lymphoma, on the other hand, have higher OR values and, therefore, exhibit significant association (Table 2). Therefore, it is thought that the *TCN 2* polymorphism rs1801198 significantly alters the circulating holotranscobalamin levels. Since TCN 2 plays a vital role in vitamin B12 metabolism, it is reasonable to suspect that the rs1801198 polymorphism may affect pathological conditions related to vitamin B12 deficiency.

*BHMT* has ~5271 polymorphisms, out of which 484 are deletions, 01 multiple base substitution, 140 are repeat variations, and 4648 are SNPs^109^. Out of these variations, three significant mutations (Gly199Ser, Glu239Arg, and Glu406His) and one polymorphism have been examined. The polymorphism is the G to A substitution (742 G > A, rs3733890), which replaces arginine by glutamine at codon 239. A study incorporating meta-analysis was performed to investigate the rs3733890 polymorphism and cancer susceptibility. It was shown that this polymorphism showed no statistically significant association with increased risk of various cancers, including head and squamous cell carcinoma, breast cancer, ovarian cancer, colorectal adenoma, and liver cancer. However, a negative association was observed in uterine cervical cancer (Table 2).

*CBS* has ~6617 polymorphisms, out of which 573 are deletions, 04 are multiple base substitutions, 1059 are repeat variations, and 4981 are SNPs^110^. Interestingly, none of these mutations have been shown to cause any significant predisposition towards any type of cancer^111^. This observation opens an avenue for future research studies focusing on understanding and identifying the frequencies of *BHMT* and *CBS* genotypes and their associations with and prevalence in different cancer types.

## I (f) CBS is associated with cancer *via* H_2_S production

H_2_S, which is a signaling molecule, is substantially involved in vasorelaxation, acting as a neuromodulator^112,113^. Recently, H_2_S has gained attention in cancer due to its cytotoxic and cytoprotective effects. It plays a key role in the bioenergetics of tumor cells and stimulates their proliferation, migration, and invasion^114–116^. In humans, CBS normally catalyzes the condensation of serine with homocysteine to produce cystathionine and water, a pivotal reaction in the trans-sulfuration pathway. In an alternative reaction, CBS can produce H_2_S *via* β-elimination and β-replacement^117^ reactions. The β-elimination reaction involves catalysis of cysteine by CBS with corresponding H_2_S production, whereas in the β-replacement mechanism, the reaction between L-cysteine and 2-mercaptoethanol enables CBS to produce H_2_S. Various clinical studies have shown that there is *CBS* overexpression and, hence, increased H_2_S production, in many cancer types, including colon, ovarian, gastric, colorectal, prostate, gastroesophageal cancer, and endometrial cell angiogenesis^118–121^ (Fiorucci, Antonelli et al.;^118^ Guo, Gai et al.;^119^ Bhattacharyya, Saha et al.;^120^ Szabo, Coletta et al. 2013; Modis, Coletta et al. 2014; Hellmich, Coletta et al.;^122^ Katsouda, Bibli et al^121^.). The role of H_2_S in cancer has been elucidated. It is known to enhance tumor growth and increase cellular proliferation by (i) stimulating cellular bioenergetics, (ii) activating proliferative, migratory, and invasive signaling pathways, and (iii) enhancing angiogenesis in tumors^122^. Other studies have demonstrated that HCT116 cells (a transformed cell line) have CBS upregulation and enhanced H_2_S production compared to nontransformed cells^123^.

ShRNA-mediated silencing of *CBS* or suppression of its activity by pharmacological means (using aminooxyacetic acid)^124^ results in reduced mitochondrial function (ATP turnover, respiratory reserve capacity, and oxygen consumption) and impaired glycolysis^112^. A clinical study of colon cancer showed reduced angiogenesis and increased growth in xenografts derived from colon cancer patients incubated in mice treated with aminooxyacetic acid^125^. Alternatively, H_2_S production induces angiogenesis in various experimental models^114^. In another development^126^, investigated the effects of S-adenosyl-L-methionine (SAM) on tumor bioenergetics^127,128^. SAM is an allosteric activator of CBS that binds to the regulatory domain of *CBS*. The study revealed that SAM enhances H_2_S production of in the HCT116 cancer cell line. Mechanistically, it is not completely known how H_2_S helps to stimulate tumor growth^129^. However, it has been argued that H_2_S serves as an autocrine stimulator during tumor proliferation. Therefore, modulation of the CBS and H_2_S levels could help limit cancer proliferation and promote its reversal.

## Conclusion

It is clear from this review that there are compelling genetic, epigenetic and environmental factors that establish a close association between disturbed Hcy metabolism and cancer. Therefore, Hcy-elevating drugs should be restrictively prescribed to cancer patients, and clinicians should closely monitor Hcy levels after chemotherapy or surgery. To date, the effects of Hcy on the growth and proliferation of tumor cells remain poorly understood. Insight into the effects of Hcy on the growth and proliferation of cancer cells would yield novel, promising strategies to curb cancer. Nevertheless, Hcy can be used as a potential tumor biomarker for a variety of cancers.

## References

[1] Mcdonald L, Bary C, Field C, Love F, Davies B (1964). Homocystinuria, thrombosis, and the blood-platelets. Lancet.

[2] Sharma GS, Kumar T, Dar TA, Singh LR (2015). Protein N-homocysteinylation: from cellular toxicity to neurodegeneration. Biochim. Biophys. Acta.

[3] Morganti M (2002). Atherosclerosis and cancer: common pathways on the vascular endothelium. Biomed. Pharmacother..

[4] Brustolin S, Giugliani R, Félix TM (2010). Genetics of homocysteine metabolism and associated disorders. Braz. J. Med. Biol. Res..

[5] Stolzenberg-Solomon RZ (1999). Pancreatic cancer risk and nutrition-related methyl-group availability indicators in male smokers. J. Natl. Cancer Inst..

[6] Seshadri S (2002). Plasma homocysteine as a risk factor for dementia and Alzheimer’s disease. N. Engl. J. Med..

[7] Vafai SB, Stock JB (2002). Protein phosphatase 2A methylation: a link between elevated plasma homocysteine and Alzheimer’s Disease. FEBS Lett..

[8] Ruud E, Holmstrøm H, Brosstad F, Wesenberg F (2006). Children with acute lymphoblastic leukaemia have high plasma levels of total homocysteine at time of diagnosis. Scand. J. Clin. Lab. Invest..

[9] Ho RCM (2011). Is high homocysteine level a risk factor for cognitive decline in elderly? a systematic review, meta-analysis, and meta-regression. Am. J. Geriatr. Psychiatry.

[10] Mattson MP, Shea TB (2003). Folate and homocysteine metabolism in neural plasticity and neurodegenerative disorders. Trends Neurosci..

[11] Obeid R, Herrmann W (2006). Mechanisms of homocysteine neurotoxicity in neurodegenerative diseases with special reference to dementia. FEBS Lett..

[12] Seshadri S (2006). Elevated plasma homocysteine levels: risk factor or risk marker for the development of dementia and Alzheimer’s disease?. J. Alzheimers Dis..

[13] Giovannucci E (1995). Alcohol, low-methionine--low-folate diets, and risk of colon cancer in men. J. Natl. Cancer Inst..

[14] Ma J (1999). A polymorphism of the methionine synthase gene: association with plasma folate, vitamin B12, homocyst(e)ine, and colorectal cancer risk. Cancer Epidemiol. Biomark. Prev..

[15] Bravatà V (2015). Controversial roles of methylenetetrahydrofolate reductase polymorphisms and folate in breast cancer disease. Int. J. Food Sci. Nutr..

[16] Kato I (1999). Serum folate, homocysteine and colorectal cancer risk in women: a nested case–control study. Br. J. Cancer.

[17] de Jong MM (2002). Low-penetrance genes and their involvement in colorectal cancer susceptibility. Cancer Epidemiol. Biomark. Prev..

[18] Matsuo K (2002). Methionine synthase reductase gene A66G polymorphism is associated with risk of colorectal cancer. Asian Pac. J. Cancer Prev..

[19] Robien K, Ulrich CM (2003). 5,10-Methylenetetrahydrofolate reductase polymorphisms and leukemia risk: a HuGE minireview. Am. J. Epidemiol..

[20] Krajinovic M (2004). Role of MTHFR genetic polymorphisms in the susceptibility to childhood acute lymphoblastic leukemia. Blood.

[21] Singal R, Ferdinand L, Das PM, Reis IM, Schlesselman JJ (2004). Polymorphisms in the methylenetetrahydrofolate reductase gene and prostate cancer risk. Int. J. Oncol..

[22] Matsuo K (2005). One-carbon metabolism related gene polymorphisms interact with alcohol drinking to influence the risk of colorectal cancer in Japan. Carcinogenesis.

[23] Mancardi D (2009). Physiological and pharmacological features of the novel gasotransmitter: hydrogen sulfide. Biochim. Biophys. Acta.

[24] Wu LL, Wu JT (2002). Hyperhomocysteinemia is a risk factor for cancer and a new potential tumor marker. Clin. Chim. Acta.

[25] Scott JM, Weir DG (1998). Folic acid, homocysteine and one-carbon metabolism: a review of the essential biochemistry. J. Cardiovasc. Risk.

[26] Selhub J (1999). Homocysteine metabolism. Annu. Rev. Nutr..

[27] Jacques PF (1996). Relation between folate status, a common mutation in methylenetetrahydrofolate reductase, and plasma homocysteine concentrations. Circulation.

[28] Schwahn BC (2003). Homocysteine-betaine interactions in a murine model of 5,10-methylenetetrahydrofolate reductase deficiency. FASEB J..

[29] Williams KT, Schalinske KL (2007). New insights into the regulation of methyl group and homocysteine metabolism. J. Nutr..

[30] Locasale JW (2013). Serine, glycine and one-carbon units: cancer metabolism in full circle. Nat. Rev. Cancer.

[31] Zhang SM (2003). Plasma folate, vitamin B6, vitamin B12, homocysteine, and risk of breast cancer. J. Natl. Cancer Inst..

[32] Siniscalchi A (2005). Increase in plasma homocysteine levels induced by drug treatments in neurologic patients. Pharmacol. Res..

[33] Smith JL, Goldsmith GA, Lawrence JD (1975). Effects of oral contraceptive steroids on vitamin and lipid levels in serum. Am. J. Clin. Nutr..

[34] Grant ECG (1983). The contraceptive pill: its relation to allergy and illness. Nutr. Health.

[35] Amatayakul K, Uttaravichai C, Singkamani R, Ruckphaopunt S (1984). Vitamin metabolism and the effects of multivitamin supplementation in oral contraceptive users. Contraception.

[36] Hjelt K, Brynskov J, Hippe E, Lundström P, Munck O (1985). Oral contraceptives and the cobalamin (Vitamin B _12_) metabolism. Acta Obstet. Gynecol. Scand..

[37] Obwegeser R, Hohlagschwandtner M, Sinzinger H (1999). Homocysteine-a pathophysiological cornerstone in obstetrical and gynaecological disorders?. Hum. Reprod. Update.

[38] Montfort WR (1990). Structure, multiple site binding, and segmental accommodation in thymidylate synthase on binding dUMP and an anti-folate. Biochemistry.

[39] Blount BC (1997). Folate deficiency causes uracil misincorporation into human DNA and chromosome breakage: implications for cancer and neuronal damage. Proc. Natl Acad. Sci. USA.

[40] Hay, R. K. M., Park, J.-G. & Gazdar, A. *Atlas of Human Tumor Cell Lines*. (Elsevier Science, Amsterdam, 1994).

[41] Crider KS, Yang TP, Berry RJ, Bailey LB (2012). Folate and DNA methylation: a review of molecular mechanisms and the evidence for Folate’s role. Adv. Nutr..

[42] Hall LE, Mitchell SE, O’Neill RJ (2012). Pericentric and centromeric transcription: a perfect balance required. Chromosom. Res..

[43] Ehrlich M (2002). DNA hypomethylation, cancer, the immunodeficiency, centromeric region instability, facial anomalies syndrome and chromosomal rearrangements. J. Nutr..

[44] Ehrlich M (2002). DNA methylation in cancer: too much, but also too little. Oncogene.

[45] Zhang D, Wen X, Wu W, Guo Y, Cui W (2015). Elevated homocysteine level and folate deficiency associated with increased overall risk of carcinogenesis: meta-analysis of 83 case-control studies involving 35,758 individuals. PLoS ONE.

[46] Stathopoulou A (2002). Molecular detection of cytokeratin-19–positive cells in the peripheral blood of patients with operable breast cancer: evaluation of their prognostic significance. J. Clin. Oncol..

[47] Refsum H (2006). The Hordaland Homocysteine Study: a community-based study of homocysteine, its determinants, and associations with disease. J. Nutr..

[48] Sun CF, Haven TR, Wu TL, Tsao KC, Wu JT (2002). Serum total homocysteine increases with the rapid proliferation rate of tumor cells and decline upon cell death: a potential new tumor marker. Clin. Chim. Acta.

[49] Akoglu B, Milovic V, Caspary WF, Faust D (2004). Hyperproliferation of homocysteinetreated colon cancer cells is reversed by folate and 5-methyltetrahydrofolate. Eur. J. Nutr..

[50] Rickles FR, Levine M, Edwards RL (1992). Hemostatic alterations in cancer patients. Cancer Metastas-. Rev..

[51] GATT A (2007). Hyperhomocysteinemia in women with advanced breast cancer. Int. J. Lab. Hematol..

[52] Green KB, Silverstein RL (1996). Hypercoagulability in cancer. Hematol. Oncol. Clin. North Am..

[53] Heit JA (2002). Relative impact of risk factors for deep vein thrombosis and pulmonary embolism: a population-based study. Arch. Intern. Med..

[54] Kakkar A, Haas S, Walsh D, Encke A (2001). Prevention of perioperative venous thromboembolism: outcome after cancer and noncancer surgery. Br. J. Surg..

[55] Zhu H (2003). Homocysteine remethylation enzyme polymorphisms and increased risks for neural tube defects. Mol. Genet. Metab..

[56] Welch GN, Loscalzo J (1998). Homocysteine and atherothrombosis. N. Engl. J. Med..

[57] Chowhan RK, Mittal S, Dar TA, Kamal MA, Singh LR (2014). Ignored avenues in alpha-synuclein associated proteopathy. CNS Neurol. Disord. Drug Targets.

[58] Sharma GS, Kumar T, Singh LR (2014). N-homocysteinylation induces different structural and functional consequences on acidic and basic proteins. PLoS ONE.

[59] Kumar T, Sharma GS, Singh LR (2016). Homocystinuria: therapeutic approach. Clin. Chim. Acta.

[60] Lentz SR (1996). Vascular dysfunction in monkeys with diet-induced hyperhomocyst (e) inemia. J. Clin. Invest..

[61] Chandrasekharan NV (2002). COX-3, a cyclooxygenase-1 variant inhibited by acetaminophen and other analgesic/antipyretic drugs: Cloning, structure, and expression. Proc. Natl Acad. Sci. USA.

[62] FitzGerald GA (2003). Parsing an enigma: the pharmacodynamics of aspirin resistance. Lancet.

[63] Goyette P (1994). Human methylenetetrahydrofolate reductase: isolation of cDNA, mapping and mutation identification. Nat. Genet..

[64] Goyette P, Frosst P, Rosenblatt DS, Rozen R (1995). Seven novel mutations in the methylenetetrahydrofolate reductase gene and genotype/phenotype correlations in severe methylenetetrahydrofolate reductase deficiency. Am. J. Hum. Genet.

[65] Kluijtmans LA (1998). Identification of four novel mutations in severe methylenetetrahydrofolate reductase deficiency. Eur. J. Hum. Genet..

[66] Weisberg I, Tran P, Christensen B, Sibani S, Rozen R (1998). A second genetic polymorphism in methylenetetrahydrofolate reductase (MTHFR) associated with decreased enzyme activity. Mol. Genet. Metab..

[67] Sibani S (2000). Characterization of six novel mutations in the methylenetetrahydrofolate reductase (MTHFR) gene in patients with homocystinuria. Hum. Mutat..

[68] Tonetti C, Amiel J, Munnich A, Zittoun J (2001). Impact of new mutations in the methylenetetrahydrofolate reductase gene assessed on biochemical phenotypes: a familial study. J. Inherit. Metab. Dis..

[69] Sharp L, Little J (2004). Polymorphisms in genes involved in folate metabolism and colorectal neoplasia: a HuGE review. Am. J. Epidemiol..

[70] Yano H (2004). Mutations of the MTHFR gene (428C > T and [458G > T + 459C > T]) markedly decrease MTHFR enzyme activity. Neurogenetics.

[71] van der Put NM (1995). Mutated methylenetetrahydrofolate reductase as a risk factor for spina bifida. Lancet.

[72] Kluijtmans LA (1996). Molecular genetic analysis in mild hyperhomocysteinemia: a common mutation in the methylenetetrahydrofolate reductase gene is a genetic risk factor for cardiovascular disease. Am. J. Hum. Genet..

[73] Brezovska-Kavrakova J (2013). Hyperhomocysteinemia and of methylenetetrahydrofolate reductase (C677T) genetic polymorphism in patients with deep vein thrombosis. Mater. Sociomed..

[74] mthfr - SNP - NCBI. Available at: https://www.ncbi.nlm.nih.gov/snp/?term=mthfr. Accessed 14 November 2018.

[75] Frosst P (1995). A candidate genetic risk factor for vascular disease: a common mutation in methylenetetrahydrofolate reductase. Nat. Genet..

[76] Weisberg IS (2001). The 1298A-- > C polymorphism in methylenetetrahydrofolate reductase (MTHFR): in vitro expression and association with homocysteine. Atherosclerosis.

[77] Etienne-Grimaldi MC (2010). Methylenetetrahydrofolate reductase (MTHFR) gene polymorphisms and FOLFOX response in colorectal cancer patients. Br. J. Clin. Pharmacol..

[78] Ma J (1997). Methylenetetrahydrofolate reductase polymorphism, dietary interactions, and risk of colorectal cancer. Cancer Res..

[79] Safarinejad MR, Shafiei N, Safarinejad S (2010). Relationship between three polymorphisms of methylenetetrahydrofolate reductase (MTHFR C677T, A1298C, and G1793A) gene and risk of prostate cancer: a case-control study. Prostate.

[80] Esteller M, Garcia A, Martinez-Palones JM, Xercavins J, Reventos J (1997). Germ line polymorphisms in cytochrome-P450 1A1 (C4887 CYP1A1) and methylenetetrahydrofolate reductase (MTHFR) genes and endometrial cancer susceptibility. Carcinogenesis.

[81] Song C, Xing D, Tan W, Wei Q, Lin D (2001). Methylenetetrahydrofolate reductase polymorphisms increase risk of esophageal squamous cell carcinoma in a Chinese population. Cancer Res..

[82] Van Guelpen BR (2006). Polymorphisms of methylenetetrahydrofolate reductase and the risk of prostate cancer: a nested case-control study. Eur. J. Cancer Prev..

[83] Johansson M (2008). Circulating concentrations of folate and vitamin B12 in relation to prostate cancer risk: results from the European Prospective Investigation into Cancer and Nutrition Study. Cancer Epidemiol. Biomark. Prev..

[84] Shannon J (2009). Folate intake and prostate cancer risk: a case-control study. Nutr. Cancer.

[85] Marugame T (2000). Methylenetetrahydrofolate reductase polymorphism and risk of colorectal adenomas. Cancer Lett..

[86] Ulrich CM, Robien K, Sparks R (2002). Pharmacogenetics and folate metabolism—a promising direction. Pharmacogenomics.

[87] Skibola CF (1999). Polymorphisms in the methylenetetrahydrofolate reductase gene are associated with susceptibility to acute leukemia in adults. Proc. Natl Acad. Sci. USA.

[88] Curtin K (2007). Genetic polymorphisms in one-carbon metabolism: associations with CpG island methylator phenotype (CIMP) in colon cancer and the modifying effects of diet. Carcinogenesis.

[89] mtrr - SNP - NCBI. Available at: https://www.ncbi.nlm.nih.gov/snp/?term=mtrr. Accessed 14 November 2018.

[90] Wilson A (1999). A common variant in methionine synthase reductase combined with low cobalamin (Vitamin B_12_) increases risk for spina bifida. Mol. Genet. Metab..

[91] Jacques PF (2003). Effects of polymorphisms of methionine synthase and methionine synthase reductase on total plasma homocysteine in the NHLBI Family Heart Study. Atherosclerosis.

[92] Goelz SE, Vogelstein B, Hamilton SR, Feinberg AP (1985). Hypomethylation of DNA from benign and malignant human colon neoplasms. Science.

[93] Alberg AJ (2000). The risk of cervical cancer in relation to serum concentrations of folate, vitamin B12, and homocysteine. Cancer Epidemiol. Biomark. Prev..

[94] Powers HJ (2005). Interaction among folate, riboflavin, genotype, and cancer, with reference to colorectal and cervical cancer. J. Nutr..

[95] Mostowska A, Hozyasz K, Jagodzinski P (2006). Maternal MTR genotype contributes to the risk of non-syndromic cleft lip and palate in the Polish population. Clin. Genet..

[96] Stolzenberg-Solomon RZ (2006). Folate intake, alcohol use, and postmenopausal breast cancer risk in the prostate, lung, colorectal, and ovarian cancer screening trial. Am. J. Clin. Nutr..

[97] Charasson V (2009). Involvement of gene polymorphisms of the folate pathway enzymes in gene expression and anticancer drug sensitivity using the NCI-60 panel as a model. Eur. J. Cancer.

[98] Ott N, Geddert H, Sarbia M (2008). Polymorphisms in methionine synthase (A2756G) and cystathionine-synthase (844ins68) and susceptibility to carcinomas of the upper gastrointestinal tract. J. Cancer Res Clin. Oncol..

[99] Hua Tao M (2009). Null results in brief DNA promoter methylation in breast tumors: no association with genetic polymorphisms in MTHFR and MTR. Cancer Epidemiol. Biomark. Prev..

[100] mthfd - SNP - NCBI. Available at: https://www.ncbi.nlm.nih.gov/snp/?term=mthfd. Accessed 14 November 2018

[101] Parle-McDermott A (2005). MTHFD1 R653Q polymorphism is a maternal genetic risk factor for severe abruptio placentae. Am. J. Med. Genet. Part A.

[102] Wang L (2007). Polymorphisms of MTHFD, plasma homocysteine levels, and risk of gastric cancer in a high-risk Chinese population. Clin. Cancer Res..

[103] Hol FA (2008). Molecular genetic analysis of the gene encoding the trifunctional enzyme MTHFD (methylenetetrahydrofolate-dehydrogenase, methenyltetrahydrofolate-cyclohydrolase, formyltetrahydrofolate synthetase) in patients with neural tube defects. Clin. Genet..

[104] Sulzyc-Bielicka V (2013). Thymidylate synthase gene polymorphism and survival of colorectal cancer patients receiving adjuvant 5-fluorouracil. Genet. Test. Mol. Biomark..

[105] tyms - SNP - NCBI. Available at: https://www.ncbi.nlm.nih.gov/snp/?term=tyms. Accessed 14 November 2018.

[106] Kawakami K, Omura K, Kanehira E, Watanabe Y (1999). Polymorphic tandem repeats in the thymidylate synthase gene is associated with its protein expression in human gastrointestinal cancers.. Anticancer. Res..

[107] tcn II - SNP - NCBI. Available at: https://www.ncbi.nlm.nih.gov/snp/?term=tcn+II. Accessed 14 November 2018.

[108] Oussalah A, Levy J, Filhine-Trésarrieu P, Namour F, Guéant JL (2017). Association of *TCN2* rs1801198 c.776G--- > C polymorphism with markers of one-carbon metabolism and related diseases: a systematic review and meta-analysis of genetic association studies. Am. J. Clin. Nutr..

[109] bhmt - SNP - NCBI. Available at: https://www.ncbi.nlm.nih.gov/snp/?term=bhmt. Accessed 14 November 2018.

[110] cbs - SNP - NCBI. Available at: https://www.ncbi.nlm.nih.gov/snp/?term=cbs. Accessed 14 November 2018.

[111] Xu X (2008). Choline metabolism and risk of breast cancer in a population-based study. FASEB J..

[112] Majtan T, Singh LR, Wang L, Kruger WD, Kraus JP (2008). Active cystathionine β-synthase can be expressed in heme-free systems in the presence of metal-substituted porphyrins or a chemical chaperone. J. Biol. Chem..

[113] Szabo C (2014). Regulation of mitochondrial bioenergetic function by hydrogen sulfide. Part I. Biochemical and physiological mechanisms. Br. J. Pharmacol..

[114] Szabó C (2007). Hydrogen sulphide and its therapeutic potential. Nat. Rev. Drug Discov..

[115] Szabó C, Papapetropoulos A (2011). Hydrogen sulphide and angiogenesis: mechanisms and applications. Br. J. Pharmacol..

[116] Whiteman M, Le Trionnaire S, Chopra M, Fox B, Whatmore J (2011). Emerging role of hydrogen sulfide in health and disease: critical appraisal of biomarkers and pharmacological tools. Clin. Sci..

[117] Kimura H (2002). Hydrogen sulfide as a neuromodulator. Mol. Neurobiol..

[118] Fiorucci S (2005). Inhibition of hydrogen sulfide generation contributes to gastric injury caused by anti-inflammatory nonsteroidal drugs. Gastroenterology.

[119] Guo H (2012). Characterization of hydrogen sulfide and its synthases, cystathionine β-Synthase and cystathionine γ-Lyase, in human prostatic tissue and cells. Urology.

[120] Bhattacharyya S (2013). Cystathionine beta-synthase (cbs) contributes to advanced ovarian cancer progression and drug resistance. PLoS ONE.

[121] Katsouda A, Bibli SI, Pyriochou A, Szabo C, Papapetropoulos A (2016). Regulation and role of endogenously produced hydrogen sulfide in angiogenesis. Pharmacol. Res..

[122] Hellmich MR, Szabo C (2015). Hydrogen sulfide and cancer. Handb. Exp. Pharmacol..

[123] Módis K (2014). Regulation of mitochondrial bioenergetic function by hydrogen sulfide. Part II. Pathophysiological and therapeutic aspects. Br. J. Pharmacol..

[124] Singh S, Padovani D, Leslie RA, Chiku T, Banerjee R (2009). Relative contributions of cystathionine β-Synthase and γ-Cystathionase to H_2_S biogenesis via alternative trans-sulfuration reactions. J. Biol. Chem..

[125] Winkler LR (2016). Population structure and genotype–phenotype associations in a collection of oat landraces and historic cultivars. Front. Plant Sci..

[126] Módis K (2014). Effect of S-adenosyl-l-methionine (SAM), an allosteric activator of cystathionine-β-synthase (CBS) on colorectal cancer cell proliferation and bioenergetics in vitro. Nitric Oxide.

[127] Singh LR, Chen X, Kožich V, Kruger WD (2007). Chemical chaperone rescue of mutant human cystathionine β-synthase. Mol. Genet. Metab..

[128] Koutmos M, Kabil O, Smith JL, Banerjee R (2010). Structural basis for substrate activation and regulation by cystathionine beta-synthase (CBS) domains in cystathionine -synthase. Proc. Natl Acad. Sci. USA.

[129] Singh LR, Gupta S, Honig NH, Kraus JP, Kruger WD (2010). Activation of mutant enzyme function in vivo by proteasome inhibitors and treatments that induce Hsp70. PLoS Genet..

[130] Mudd SH (2000). Homocysteine and its disulfide derivatives. Arterioscler. Thromb. Vasc. Biol..

[131] Mudd SH (1985). The natural history of homocystinuria due to cystathionine beta-synthase deficiency. Am. J. Hum. Genet.

[132] Wilcken DE, Wilcken B (1976). The pathogenesis of coronary artery disease. A possible role for methionine metabolism. J. Clin. Invest..

[133] Eberhardt RT (2000). Endothelial dysfunction in a murine model of mild hyperhomocyst (e) inemia. J. Clin. Invest..

[134] Harker LA, Harlan JM, Ross R (1983). Effect of sulfinpyrazone on homocysteine-induced endothelial injury and arteriosclerosis in baboons. Circ. Res..

[135] Quadri P (2004). Homocysteine, folate, and vitamin B-12 in mild cognitive impairment, Alzheimer disease, and vascular dementia. Am. J. Clin. Nutr..

[136] McIlroy SP, Dynan KB, Lawson JT, Patterson CC, Passmore AP (2002). Moderately elevated plasma homocysteine, methylenetetrahydrofolate reductase genotype, and risk for stroke, vascular dementia, and Alzheimer disease in Northern Ireland. Stroke.

[137] Rosenquist TH, Ratashak SA, Selhub J (1996). Homocysteine induces congenital defects of the heart and neural tube: effect of folic acid. Proc. Natl Acad. Sci. USA.

[138] Hobbs CA, Cleves MA, Melnyk S, Zhao W, James SJ (2005). Congenital heart defects and abnormal maternal biomarkers of methionine and homocysteine metabolism. Am. J. Clin. Nutr..

[139] McDowell IF, Lang D (2000). Homocysteine and endothelial dysfunction: a link with cardiovascular disease. J. Nutr..

[140] Refsum H, Ueland PM, Nygard O, Vollset SE (1998). Homocysteine and cardiovascular disease. Annu Rev. Med.

[141] Martignoni E (2007). Homocysteine and Parkinson’s disease: a dangerous liaison?. J. Neurol. Sci..

[142] Lamberti P (2005). Effects of levodopa and COMT inhibitors on plasma homocysteine in Parkinson’s disease patients. Mov. Disord..

[143] Moustafa AA, Hewedi DH, Eissa AM, Frydecka D, Misiak B (2014). Homocysteine levels in schizophrenia and affective disorders-focus on cognition. Front. Behav. Neurosci..

[144] Muntjewerff JW, Kahn RS, Blom HJ, den Heijer M (2006). Homocysteine, methylenetetrahydrofolate reductase and risk of schizophrenia: a meta-analysis. Mol. Psychiatry.

[145] Leblhuber F (2000). Hyperhomocysteinemia in dementia. J. Neural Transm..

[146] Shea, T. B. & Rogers, E. Homocysteine and dementia. *N Engl J Med* **346**, 2007–2008 (2002). 10.1056/NEJM200206203462514 12075065

[147] Tiemeier H (2002). Vitamin B_12_, folate, and homocysteine in depression: the Rotterdam Study. Am. J. Psychiatry.

[148] Almeida OP (2008). Homocysteine and depression in later life. Arch. Gen. Psychiatry.

[149] Janula A (2005). Homocysteine and diabetes. Wiad. Lek. (Wars., Pol. 1960).

[150] Baliga BS, Reynolds T, Fink LM, Fonseca VA (2000). Hyperhomocysteinemia in type 2 diabetes mellitus: cardiovascular risk factors and effect of treatment with folic acid and pyridoxine. Endocr. Pract..

[151] Gueant JL (2005). Homocysteine and related genetic polymorphisms in Down’s syndrome IQ. J. Neurol. Neurosurg. Psychiatry.

[152] Schuh S (1984). Homocystinuria and megaloblastic anemia responsive to vitamin B12 therapy. An inborn error of metabolism due to a defect in cobalamin metabolism. N. Engl. J. Med.

[153] Wong WY (1999). Nonsyndromic orofacial clefts: association with maternal hyperhomocysteinemia. Teratology.

[154] Brenton DP, Dow CJ, James JIP, Hay RL, Wynne-Davies R (1972). Homocystinuria and Marfan’s syndrome. Bone Jt. J..

[155] Paynter RA, Hankinson SE, Hunter DJ, De Vivo I (2004). No association between MTHFR 677 C->T or 1298 A--- > C polymorphisms and endometrial cancer risk. Cancer Epidemiol. Biomark. Prev..

[156] Shujuan Y, Jianxing Z, Xin-Yue C (2013). Methylenetetrahydrofolate reductase genetic polymorphisms and esophageal squamous cell carcinoma susceptibility: a meta-analysis of case-control studies. Pak. J. Med. Sci..

[157] He L, Shen Y (2017). MTHFR C677T polymorphism and breast, ovarian cancer risk: a meta-analysis of 19,260 patients and 26,364 controls. Onco. Targets Ther..

[158] Li XL, Xu JH (2012). MTHFR polymorphism and the risk of prostate cancer: a meta-analysis of case–control studies. Prostate Cancer Prostatic Dis..

[159] Chen J (1996). A methylenetetrahydrofolate reductase polymorphism and the risk of colorectal cancer. Cancer Res..

[160] Chen J (1998). A prospective study of methylenetetrahydrofolate reductase and methionine synthase gene polymorphisms, and risk of colorectal adenoma. Carcinogenesis.

[161] Ulvik A (2004). Colorectal cancer and the methylenetetrahydrofolate reductase 677C --- > T and methionine synthase 2756A -> G polymorphisms: a study of 2,168 case-control pairs from the JANUS cohort. Cancer Epidemiol. Biomark. Prev..

[162] Qin YT (2014). Association between MTHFR polymorphisms and acute myeloid leukemia risk: a meta-analysis. PLoS ONE.

[163] Haghighi MM (2008). Association between the 1793G---- > A MTHFR polymorphism and sporadic colorectal cancer in Iran. Asian Pac. J. Cancer Prev..

[164] Fang DH, Ji Q, Fan CH, An Q, Li J (2014). Methionine synthase reductase A66G polymorphism and leukemia risk: evidence from published studies. Leuk. Lymphoma.

[165] Wang P, Li S, Wang M, He J, Xi S (2017). Association of MTRR A66G polymorphism with cancer susceptibility: evidence from 85 studies. J. Cancer.

[166] Wu PP, Tang RN, An L (2015). A meta-analysis of MTRR A66G polymorphism and colorectal cancer susceptibility. J. Buon..

[167] Yuan LJ (2012). Polymorphisms of tumor-related genes IL-10, PSCA, MTRR and NOC3L are associated with the risk of gastric cancer in the Chinese Han population. Cancer Epidemiol..

[168] Yoo JY (2012). Association study between folate pathway gene single nucleotide polymorphisms and gastric cancer in Koreans. Genom. Inform..

[169] Wu X (2014). Plasma homocysteine levels and genetic polymorphisms in folate metablism are associated with breast cancer risk in chinese women. Hered. Cancer Clin. Pract..

[170] Zhao Y (2013). Lack of association between methionine synthase A2756G polymorphism and digestive system cancer risk: evidence from 39327 subjects. PLoS ONE.

[171] Chen K (2006). Association between genetic polymorphisms in folate metabolic enzyme genes and colorectal cancer: a nested case-control study. Zhonghua Zhong Liu Za Zhi.

[172] Shi Q (2005). Polymorphisms of methionine synthase and methionine synthase reductase and risk of lung cancer: a case-control analysis. Pharm. Genom..

[173] Cui LH (2012). Folate metabolism-related gene polymorphisms and susceptibility to primary liver cancer in North China. Med. Oncol..

[174] Semmler A, Simon M, Moskau S, Linnebank M (2006). The methionine synthase polymorphism c.2756A---- > G alters susceptibility to glioblastoma multiforme. Cancer Epidemiol. Biomark. Prev..

[175] Zhang FF (2007). Genetic polymorphisms in folate metabolism and the risk of stomach cancer. Cancer Epidemiol. Biomark. Prev..

[176] Suzuki T (2008). Alcohol drinking and one-carbon metabolism-related gene polymorphisms on pancreatic cancer risk. Cancer Epidemiol. Biomark. Prev..

[177] Zhang H, Ma H, Li L, Zhang Z, Xu Y (2013). Association of methylenetetrahydrofolate dehydrogenase 1 polymorphisms with cancer: a meta-analysis. PLoS ONE.

[178] Cui Y, Jing Y, Sun Z (2014). Lack of association between MTHFD1 G401A polymorphism and ovarian cancer susceptibility. Tumor Biol..

[179] da Silva LMRB (2012). MTHFD1 G1958A, BHMT G742A, TC2 C776G and TC2 A67G polymorphisms and head and neck squamous cell carcinoma risk. Mol. Biol. Rep..

[180] Xu X (2008). B-vitamin intake, one-carbon metabolism, and survival in a population-based study of women with breast cancer. Cancer Epidemiol. Biomark. Prev..

[181] Mostowska A, Myka M, Lianeri M, Roszak A, Jagodziński PP (2011). Folate and choline metabolism gene variants and development of uterine cervical carcinoma. Clin. Biochem..

[182] Pawlik P (2012). Folate and choline metabolism gene variants in relation to ovarian cancer risk in the Polish population. Mol. Biol. Rep..

[183] Hazra A (2007). Twenty-four non-synonymous polymorphisms in the one-carbon metabolic pathway and risk of colorectal adenoma in the Nurses’ Health Study. Carcinogenesis.

[184] Chang SC (2014). Single nucleotide polymorphisms of one-carbon metabolism and cancers of the esophagus, stomach, and liver in a Chinese population. PLoS ONE.

[185] Koushik A (2006). Nonsynonymous polymorphisms in genes in the one-carbon metabolism pathway and associations with colorectal cancer. Cancer Epidemiol. Biomark. Prev..

[186] Kurzwelly D (2010). Genetic variants of folate and methionine metabolism and PCNSL incidence in a German patient population. J. Neurooncol..

[187] Gao CM (2004). Polymorphisms in thymidylate synthase and methylenetetrahydrofolate reductase genes and the susceptibility to esophageal and stomach cancer with smoking. Asian Pac. J. Cancer Prev..

